# Statistical aspects of omics data analysis using the random compound covariate

**DOI:** 10.1186/1752-0509-6-S3-S11

**Published:** 2012-12-17

**Authors:** Pei-Fang Su, Xi Chen, Heidi Chen, Yu Shyr

**Affiliations:** 1 Center for Quantitative Sciences, Vanderbilt University, Nashville, TN, USA; 2 Department of Biostatistics, Vanderbilt University, Nashville, TN, USA

## Abstract

**Background:**

Dealing with high dimensional markers, such as gene expression data obtained using microarray chip technology or genomics studies, is a key challenge because the numbers of features greatly exceeds the number of biological samples. After selecting biologically relevant genes, how to summarize the expression of selected genes and then further build predicted model is an important issue in medical applications. One intuitive method of addressing this challenge assigns different weights to different features, subsequently combining this information into a single score, named the compound covariate. Investigators commonly employ this score to assess whether an association exists between the compound covariate and clinical outcomes adjusted for baseline covariates. However, we found that some clinical papers concerned with such analysis report bias p-values based on flawed compound covariate in their training data set.

**Results:**

We correct this flaw in the analysis and we also propose treating the compound score as a random covariate, to achieve more appropriate results and significantly improve study power for survival outcomes. With this proposed method, we thoroughly assess the performance of two commonly used estimated gene weights through simulation studies. When the sample size is 100, and censoring rates are 50%, 30%, and 10%, power is increased by 10.6%, 3.5%, and 0.4%, respectively, by treating the compound score as a random covariate rather than a fixed covariate. Finally, we assess our proposed method using two publicly available microarray data sets.

**Conclusion:**

In this article, we correct this flaw in the analysis and the propose method, treating the compound score as a random covariate, can achieve more appropriate results and improve study power for survival outcomes.

## Introduction

### High-dimensional omics data

Personalized medicine is expected to enable a more predictive discipline, in which therapies are targeted toward the molecular constitution of individual patients and their disease; thus, molecular biomarkers are widely expected to revolutionize the current practice of medicine. For example, the progress of genomics has made it possible to evaluate molecular signatures to predict cancer metastasis [1,2]. Various technological breakthroughs have led to a plethora of high-dimensional omics data to support personalized medicine, and these data have a common characteristic: the numbers of features greatly exceeds the number of biological samples. Because biological phenomena are the result of sets of features (e.g., concerted expression of multiple genes), the analysis of a group of related features (e.g., genes) may be more effective and may provide more directly interpretable results than the analysis of individual genes.

As high-dimensional omics research has advanced, the compound covariate (or compound score) has generally been held as a simpler and more straightforward approach. After selecting biologically relevent genes in training cohort, such a score is often a useful device in medical applications to define the information contained in a single set of data and to summarize the association of a set of variables with disease. Tukey [3] first advocated the use of compound covariates in the clinical trial setting. To develop a compound score, the individual covariates are summed; the association between such a compound covariate and outcome then is evaluated via regression analysis. Tomasson [4] used a compound score for binary outcomes, via fitting a logistic regression. Later, Hedenfalk [5] successfully applied the compound covariate method to class prediction analysis for breast cancer data. Because the use of the compound covariate is intuitive and seems useful, many other leading researchers also have applied this method for analyzing omics data sets [6-9].

### Problem statements

A compound covariate is a linear combination of the basic covariates being studied, with each covariate having its own coefficient or weight. For survival outcomes, a commonly used scheme is to 1) compute the univariate Cox regression [10] for each gene of interest, 2) assign a weight to each gene (typically, the estimated regression coefficients or Wald statistics from the univariate Cox regressions), and 3) combine the weighted genes in a linear model that incorporates gene expression levels in each sample. This method of modeling weighted genes is believed to reflect the importance of each individual gene to the outcome; the higher the weight assigned, the more significant a particular gene is.

**Figure 1:**
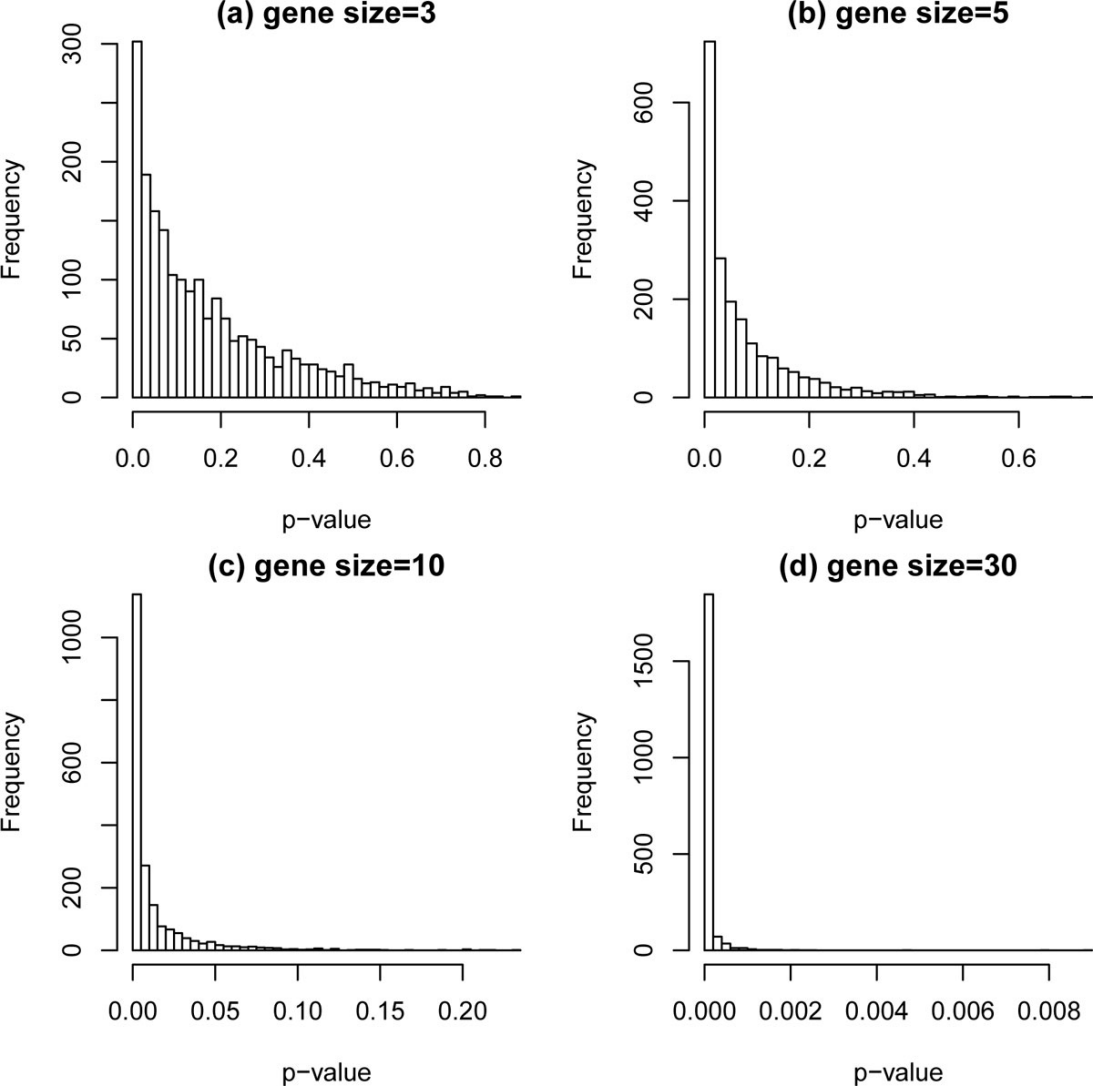
**P-value distribution under the null hypothesis with nominal level 0.05**.

However, the linear combination of the group of genes, with each gene having its own estimated weight, should not be treated as an observed covariate or fixed covariate. Because selected weights are estimated through computing the univariate Cox regression of each individual gene, the compound "covariate" should be treated as a random covariate that includes estimated error. Besides, for the purpose of assessing whether an association exists between the compound covariate and survival outcomes, Cox regression is typically used to evaluate the significance level of the parameter of the compound score. However, bias concerns arise when the same data set, training cohort, is used for a double purpose: to construct the compound covariate and then to test it. This framework results in an over-fitting problem. As shown in Figure 1, we simulate 50 observations with 3, 5, 10, and 30 non-causal genes used to create a compound covariate. The Cox regression model is then used to test whether an association exists between the compound scores and survival outcomes in the same dataset. The distribution of p-values should be uniform in the interval [0, 1]. In our simulation, however, p-values are concentrated around zero, especially as the number of genes increases. This demonstrates that type I error rates are inflated and consequently not controlled. Obviously, double using the training cohort casue overfitting problem and bias p-values arised. We found that some medical papers report inappropriate p-values for testing training cohort data [11,6]. Although the proposed bias p-values in their training cohort do not affact their final research results inferred from another independent testing data set, these observations motive us to study a more appropriate testing procedure for the compound covariate if the investagtors whish to report a testing result in the training cohort.

In this paper, we first contend the compound covariate should be treated as a random observation. Our idea is based on that proposed by Prentice [12], who analyzed covariates with measurement error and used a partial likelihood function technique to infer whether the parameter for the covariate was significant. In addition, if a training data set is used for a double purpose (i.e., to construct the compound covariate and then to test it), the resulting over-fitting means the p-value is not reliable when testing the regression parameter. Therefore, we use a 2-fold method (e.g., [13,14]), splitting all observations in the training cohort into two parts, one part for assigning gene weights, and another part for testing the regression parameter through a partial likelihood score test. The remainder of this paper is organized as follows: We outline creation of the compound score using a random covariate approach. Then, we investigate the accuracy of the asymptotic distribution of the proposed tests. We thoroughly assess the performance of two commonly used estimated weights, "estimated coefficient" and "Wald statistic", for the Cox proportional hazards model. Finally, we illustrate the implementation of the proposed methods through two real data sets, and offer concluding remarks.

## The proposed method

### The compound covariate

In this section, we formally define some notations for compound covariate and introduce a procedure to identify whether a set of genes is truly associated with survival times in the training cohort. Let *T_j _*denote the survival time and *C_j _*denote the censoring time independent of *T_j _*for the *j*th patient, with *j *= 1, 2, ..., *n*. When some observations are right-censored, one can observe only random variables *X_j _*= min(*T_j_, C_j_*) and *δ_j _*= *I*(*T_j _*≤ *C_j_*), where *I*(*A*) is an indicator function of event *A*, assuming the value 1 if event *A *occurs and the value 0 otherwise. Let *x_j _*= (*x*_*j*1_, *x*_*j*2_, ..., *x_jp_*) be the standardized selected gene's intensity in the *j*th patient, where *p *is the size of the gene set. For creating the compound covariate, one first fits the univariate Cox regression model for each individual gene, that is,

(1) $$h\left(t|x_{k}\right)=h_{0k}\left(t\right)\text{exp}\left(x_{k}\beta_{k}\right),k=1,2,\ldots ,p$$

where *h*_0*k*_(*t*) is a baseline hazard function of each gene *k*, and *β_k _*is a corresponding parameter to be estimated. Let ${\hat{\beta }}_{1},{\hat{\beta }}_{2},\ldots ,{\hat{\beta }}_{p}$ be the estimators of *β*_1_, *β*_2_, ..., *β_p_*, and ${ŵ}_{1},{ŵ}_{2},\ldots ,{ŵ}_{p}$ be the Wald statistics obtained from (1). A compound covariate commonly used by clinicians is defined as

$$z_{j}=\overset{p}{\underset{k=1}{\sum }}x_{jk}{\hat{\beta }}_{k},$$

or another possible weighting policy depends on Wald statistics,

$$z_{j}=\overset{p}{\underset{k=1}{\sum }}x_{jk}{ŵ}_{k}\text{sign}\left({\hat{\beta }}_{k}\right),$$

for each patient *j, j *= 1, 2, ..., *n*. From the perspective of biology, the weighting policy is believed to reflect the importance of each individual gene to survival outcome, the higher the weight, the more important the gene is. In other words, the score can be regarded as a condensed index, representing the collective effects of gene expression.

To identify whether this gene expression pattern is truly associated with survival in training cohort, investigators prefer to use Cox regression analysis. That is, after fitting model (1), they construct

$$h\left(t\;|\;z\right)=h_{0}\left(t\right)\text{exp}\left(\gamma_{0}z\right)$$

where *h*_0_(*t*) is a baseline hazard function and γ_0 _is a corresponding parameter for the compound covariate *z*; they then use the same data set to test the null hypothesis *H*_0 _:*γ*_0 _= 0. Under the null hypothesis, however, the method results in uncontrolled type I error, because the training data set has been used twice, both for building the model and for testing the regression parameter. If independent data are available, carry ${\hat{\beta }}_{1},{\hat{\beta }}_{2},\ldots ,{\hat{\beta }}_{p}$ from training data set and test on another independent data set is possible, allowing unbiased model validation to prevent over-fitting. However, if investagtors whish to report a testing result in the training cohort, an alternative, though less optimal, study design is using k-fold method or split the training cohort data randomly (2-fold), with 50% of the data being assigned to develop the score, compound covariate, and 50% to evaluate its performance. The limitation of this approach is that it requires a relatively large sample size. With this method, Kaplan-Meier survival curves [15] for the two sets should be examined to ensure no significant difference by the random selection of those two sets from training cohort data.

### Cox regression with a random compound covariate

The measuring mechanism makes the compound covariate an estimation and not a fixed observable. Naturally, such a covariate should be treated as a random covariate, and the variance of each score needs to be taken into account. To fit a Cox regression model with a random covariate, we use the idea advocated by Prentice, which presents the Cox model as a multiplicative hazards model, with a relative risk at time *t*,

(2) $$E\left[\text{exp}\left(\gamma_{0}z\right)\right].$$

This is a weighted average of a possible relative risk given the covariate *z*. The Cox regression model then can be written as

$$h\left(t|z\right)=h_{0}\left(t\right)E\left[\text{exp}\left(\gamma_{0}z\right)\right].$$

Because omics data sets involve a large number of features, we assume the distributions of the scores follow normal distribution. That is, for each patient *j*, assuming *z_j _*is drawn from a normal distribution with mean *μ_j _*and variance $\sigma_{j}^{2}$, (2) can be derived as

$$\text{exp}\left(\gamma_{0}\mu_{j}+\frac{1}{2}\sigma_{j}^{2}\gamma_{0}^{2}\right).$$

Thus, a quadratic term, $\sigma_{j}^{2}\gamma_{0}^{2}/2$, is added to the relative risk function, accounting for the variance in the random covariate. In addition, with both random covariates *z *(in this case, the compound covariate) and observed covariates **w **with dimension *d *(in this case, the clinical variable), it is easy to incorporate the fixed covariate effects into the Cox model, as:

$$h\left(t|z,w\right)=h_{0}\left(t\right)E\left[\text{exp}\left(\gamma_{0}z+\gamma_{1}^{\text{T}}w\right)\right]\text{,}$$

where *γ*_0 _is the parameter for the compound covariate, $\gamma_{1}$ is the corresponding parameters for fixed observations and $\gamma_{1}^{\text{T}}$ is the transpose of $\gamma_{1}$.

### A partial likelihood function and score test

Suppose now that *t*_1 _< ... <*t_l _*are the ordered distinct survival times in the sample, and let *R*(*t_i_*) and *F*(*t_i_*) denote the risk set prior to *t_i _*and the set of subjects failing at *t_i_*, respectively. The partial likelihood function is:

$$L\left(\gamma \right)=\overset{l}{\underset{i=1}{\prod }}\frac{\prod_{j\in F\left(t_{i}\right)}E\left[\text{exp}\left(\gamma_{\text{0}}z+\gamma_{1}^{\text{T}}w\right)\right]}{\sum_{j\in R\left(t_{i}\right)}E{\left[\text{exp}\left(\gamma_{0}z+\gamma_{1}^{\text{T}}w\right)\right]}^{m_{i}}},$$

where *m_i _*is the number of failures at time *t_i_*(*i *= 1, 2,...*l*). Let $a=E\left[\text{exp}\left\{\gamma_{0}z+\gamma_{1}^{\text{T}}w\right\}\right]$, b=∂a/∂γ and c=∂b/∂γ where $\gamma ={\left[\gamma_{0},\;\gamma_{1}^{\text{T}}\right]}^{\text{T}}$ (The explicit forms of *a, b *and *c *are shown in Additional file 1). The score statistic then can be derived as

(3) $$v=\frac{\partial \text{log}L\left(\gamma \right)}{\partial \gamma }=\overset{l}{\underset{i=1}{\sum }}\left(\underset{j\in F\left(t_{i}\right)}{\sum }\frac{b_{ij}}{a_{ij}}-m_{i}\frac{\sum_{j\in R\left(t_{i}\right)}b_{ij}}{\sum_{j\in R\left(t_{i}\right)}a_{ij}}\right)$$

with the observed information matrix ***V***

(4) $$\begin{array}{ccc} -\frac{\partial^{2}\text{log}L\left(\gamma \right)}{\partial \gamma^{2}}=\overset{l}{\underset{i=1}{\sum }} & m_{i}\frac{\sum_{j\in R\left(t_{i}\right)}c_{ij}}{\sum_{j\in R\left(t_{i}\right)}a_{ij}}-\overset{l}{\underset{i=1}{\sum }}m_{i}{\left(\frac{\sum_{j\in R\left(t_{i}\right)}b_{ij}}{\sum_{j\in R\left(t_{i}\right)}a_{ij}}\right)}^{2} & \\ & -\overset{l}{\underset{i=1}{\sum }}\underset{j\in F\left(t_{i}\right)}{\sum }\left(\frac{c_{ij}}{a_{ij}}-\frac{b_{ij}^{2}}{a_{ij}^{2}}\right). \end{array}$$

Consequently, under the null hypothesis $H_{0}:\gamma =0$, the partial likelihood score test *v*^T^***V***^-1 ^*v *will have an asymptotic $\chi_{d+1}^{2}$ distribution when ***V ***is nonsingular. In addition, (3) can be used in a standard manner for γ  estimation. In practice, we can calculate a p-value by replacing *μ_j _*with *z_j _*and $\sigma_{j}^{2}$ with Var(*z_j_*) where

$$\text{Var}\left(z_{j}\right)=\overset{p}{\underset{k=1}{\sum }}\text{Var}\left(x_{jk}{\hat{\beta }}_{k}\right)=\overset{p}{\underset{k=1}{\sum }}x_{jk}^{2}s_{\beta_{k}}^{2},$$

is derived based on approximation and $s_{\beta k}^{2}$ is the variance estimation of ${\hat{\beta }}_{k}$ by using estimated coefficient as weight or

$$\text{Var}\left(z_{j}\right)=\overset{p}{\underset{k=1}{\sum }}\text{Var}\left(x_{jk}{ŵ}_{k}\text{sign}\left({\hat{\beta }}_{k}\right)\right)=3\overset{p}{\underset{k=1}{\sum }}x_{jk}^{2}$$

by using Wald statistics as weight. We show the derivation in more detail in Additional file 2.

### Multiple gene sets

Further, we extended the compound covariate to multiple gene sets. If there are *q *given independent gene sets for the *j*th patient, $x_{j}^{\left(1\right)},x_{j}^{\left(2\right)},\ldots ,x_{j}^{\left(q\right)}$, where $x_{j}^{\left(s\right)}=\left(x_{1j}^{\left(s\right)},\;x_{2j}^{\left(s\right)},\;\ldots ,\;x_{p_{s}j}^{\left(s\right)}\right)$, *s *= 1, 2, ..., *q *, the compound covariates can be written as vector

$$z_{j}=\left(z_{j}^{\left(1\right)},z_{j}^{\left(2\right)},\ldots ,z_{j}^{\left(s\right)}\right)=\left(\overset{p_{1}}{\underset{k=1}{\sum }}x_{jk}^{\left(1\right)}{\hat{\beta }}_{k},\overset{p_{2}}{\underset{k=1}{\sum }}x_{jk}^{\left(2\right)}{\hat{\beta }}_{k},\ldots ,\overset{p_{s}}{\underset{k=1}{\sum }}x_{jk}^{\left(s\right)}{\hat{\beta }}_{k}\right),\text{or}$$

$$z_{j}=\left(\overset{p_{1}}{\underset{k=1}{\sum }}x_{jk}^{\left(1\right)}{ŵ}_{k}\text{sign}\left({\hat{\beta }}_{k}\right),\overset{p_{2}}{\underset{k=1}{\sum }}x_{jk}^{\left(2\right)}{ŵ}_{k}\text{sign}\left({\hat{\beta }}_{k}\right),\ldots ,\overset{p_{s}}{\underset{k=1}{\sum }}x_{jk}^{\left(s\right)}{ŵ}_{k}\text{sign}\left({\hat{\beta }}_{k}\right)\right)$$

where *p_s _*is the number of genes of the *s*th gene set. Then, the partial likelihood function can be written as

$$L\left(\gamma \right)=\overset{l}{\underset{i=1}{\prod }}\frac{\prod_{l\in F\left(t_{i}\right)}E\left[\text{exp}\left(\gamma_{0}^{\text{T}}z+\gamma_{1}^{\text{T}}w\right)\right]}{\sum_{l\in R\left(t_{i}\right)}E{\left[\text{exp}\left(\gamma_{0}^{\text{T}}z+\gamma_{1}^{\text{T}}w\right)\right]}^{m_{i}}}.$$

Let $a=E\left[\text{exp}\left(\gamma_{0}^{\text{T}}z+\gamma_{1}^{\text{T}}w\right)\right]$, b=∂a/∂γ and c=∂b/∂γ, where $\gamma ={\left[\gamma_{0}^{\text{T}},\gamma_{1}^{\text{T}}\right]}^{\text{T}}$. The score statistic and the observed information matrix can be further derived as (3) and (4) as well. Consequently, under the null hypothesis $H_{0}:\gamma =0$, the partial likelihood score test *v*^T^***V***^-1 ^*v *has an asymptotic $\chi_{d+q}^{2}$ distribution when ***V ***is nonsingular. If we reject the null hypothesis, we can conclude that the covariate vector is associated with survival time.

### Simulation results

To assess the performance of the proposed testing procedure for compound covariate, we conducted simulation studies under various scenarios to study type I error rate and power. For the scenario of split training data set as two parts and the consideration of compound scores as random covariates, we denoted the compound score using $\hat{\beta }$ as a weight function as *SRC_B_*, and the compound score using the Wald statistic as a weight function as *SRC_W_*. The corresponding notations, *SC_B _*and *SC_W_*, refer to split data but without treating the compound covariate as a random covariate (i.e., typical Cox regression [10]). The notations *DC_B _*and *DC_W _*refer to compound scores with double use of the training data set, both for building the model and then testing for the same data set. Assume there are *p *selected genes in a gene set. Gene expression data were generated from a multivariate normal distribution with mean vector ***β ***= [*β*_1_, *β*_2_, ..., *β_p_*]^T^, and variance-covariance matrix equal to the identity matrix for *n *cases. Generated survival times were associated with gene expression via a proportional hazard model, exp(*x*^T^***β***). All tests with nominal significance level 0.05 were applied and empirical rejection probability was obtained based on 2000 simulation runs.

For comparing empirical type I error rates, the value of ***β ***was set to 0. The total sample size was set to 50, 75, or 100. After gene selection process, we assume the total number of disease relative genes was set to 10, 30, 50, or 70. Censoring times (denoted as cen.) were generated from an exponential distribution, and the overall censoring fraction in either setup was fixed at 10% or 40%. Table 1 shows the empirical type I error rates. As shown, the proposed procedure for *SRC_B _*and *SRC_W _*preserve reasonable type I error rates. The methods *DC_B _*and *DC_W_*, however, which make double use of the data set, do not control type I error. Note that we do not show type I error for *SC_B _*and *SC_W _*methods, because these methods are typical Cox regression.

**Table 1:** Empirical type I error rates

Method	*n*	**cen**.	The total number of genes			
			10	30	50	70
*SRC_B_*	50	10%	0.052	0.057	0.051	0.048
		40%	0.041	0.047	0.045	0.046
	75	10%	0.052	0.048	0.045	0.046
		40%	0.044	0.046	0.050	0.046
	100	10%	0.056	0.049	0.052	0.052
		40%	0.045	0.044	0.048	0.050
*SRC_W_*	50	10%	0.058	0.046	0.052	0.050
		40%	0.034	0.046	0.036	0.043
	75	10%	0.046	0.042	0.051	0.051
		40%	0.044	0.038	0.044	0.040
	100	10%	0.051	0.046	0.048	0.060
		40%	0.044	0.041	0.046	0.048

*DC_B_*	50	10%	0.937	1.000	1.000	1.000
		40%	0.910	1.000	1.000	1.000
	75	10%	0.944	1.000	1.000	1.000
		40%	0.946	1.000	1.000	1.000
	100	10%	0.957	1.000	1.000	1.000
		40%	0.952	1.000	1.000	1.000
*DC_W_*	50	10%	0.926	1.000	1.000	1.000
		40%	0.916	1.000	1.000	1.000
	75	10%	0.920	1.000	1.000	1.000
		40%	0.936	1.000	1.000	1.000
	100	10%	0.929	1.000	1.000	1.000
		40%	0.933	1.000	1.000	1.000

Empirical type I error rates for comparing *SRC_B_, SRC_W _DC_B _*and *DC_W _*methods. The value of *β *was set to 0.

Because type I error rates are preserved for both the *SRC_B_*/*SRC_W _*and *SC_B_*/*SC_W _*methods, we compared the power under each method in Table 2. In this simulation, censoring times were also generated from an exponential distribution, and the overall censoring fraction in either setup was fixed at 10%, 30%, or 50%. We then simulated 30 total genes in one gene set under two different scenarios. The first scenario considers 30 disease related genes. We designed two different levels of effect, strong effect and low effect, as

**Table 2:** Power comparison under two different scenario

*n*	**cen**.	Scenarios 1				Scenarios 2			
		*SRC_B_*	*SC_B_*	*SRC_W_*	*SC_W_*	*SRC_B_*	*SC_B_*	*SRC_W_*	*SC_W_*
Strong effect: ***β ***= [*β*_1_, *β*_2_, ..., *β*_30_]^T ^= [1, 1, ..., 1]^T^									
50	10%	0.757	0.742	0.675	0.650	0.600	0.599	0.723	0.692
	30%	0.624	0.580	0.536	0.490	0.480	0.422	0.546	0.505
	50%	0.448	0.350	0.381	0.312	0.350	0.250	0.359	0.294
75	10%	0.960	0.956	0.907	0.902	0.876	0.870	0.944	0.942
	30%	0.883	0.864	0.828	0.766	0.783	0.771	0.875	0.822
	50%	0.758	0.626	0.690	0.526	0.607	0.494	0.694	0.580
100	10%	0.998	0.997	0.985	0.982	0.974	0.973	0.996	0.995
	30%	0.982	0.974	0.948	0.917	0.940	0.905	0.966	0.955
	50%	0.928	0.846	0.868	0.730	0.806	0.695	0.883	0.802
Low effect: ***β ***= [*β*_1_, *β*_2_, ..., *β*_30_]^T ^= [0.5, 0.5, ..., 0.5]^T^									
50	10%	0.666	0.625	0.594	0.576	0.266	0.242	0.326	0.305
	30%	0.498	0.487	0.440	0.430	0.206	0.165	0.224	0.214
	50%	0.362	0.285	0.328	0.244	0.144	0.122	0.162	0.124
75	10%	0.930	0.924	0.859	0.850	0.492	0.466	0.574	0.570
	30%	0.824	0.756	0.756	0.688	0.370	0.325	0.432	0.421
	50%	0.642	0.553	0.571	0.469	0.263	0.206	0.312	0.224
100	10%	0.992	0.990	0.964	0.950	0.662	0.654	0.796	0.792
	30%	0.959	0.944	0.918	0.850	0.558	0.505	0.652	0.594
	50%	0.852	0.760	0.802	0.654	0.412	0.319	0.472	0.370

We compared the power under each method *SRC_B_*/*SRC_W _*and *SC_B_*/*SC_W_*. The first scenario considers 30 disease related genes. The second scenario considers 30 disease related genes, with the other 27 genes considered no effect.

$$\beta ={\left[\beta_{1},\beta_{2},\ldots ,\beta_{30}\right]}^{\text{T}}={\left[1,1,\ldots ,1\right]}^{\text{T}},$$

$$\beta ={\left[\beta_{1},\beta_{2},\ldots ,\beta_{30}\right]}^{\text{T}}={\left[0.5,0.5,\ldots ,0.5\right]}^{\text{T}},$$

respectively. The second scenario considers 3 disease related genes, with the other 27 genes considered "noise" (i.e., no effect). Strong effect and low effect in this case were set as

$$\beta ={\left[\beta_{1},\beta_{2},\ldots ,\beta_{30}\right]}^{\text{T}}={\left[1,1,1,0\ldots ,0\right]}^{\text{T}},$$

$$\beta ={\left[\beta_{1},\beta_{2},\ldots ,\beta_{30}\right]}^{\text{T}}={\left[0.5,0.5,0.5,\ldots ,0\right]}^{\text{T}}.$$

Results are shown in Table 2.

For scenario 1, all 30 genes have effects. As expected, the power of the tests increases with increase in total sample size and gene effect, but decreases as the censoring proportion grows. Under the first scenario, the power of the *SRC_B _*method is always better than that of *SC_B_*, and *SRC_W _*is always better than *SC_W_*. This result indicates that treating the compound score as a random covariate yields higher power than treating the score as a fixed covariate. When the sample size is 100, the power average increases 10.6, 3.5, and 0.4 percentage points for 50%, 30%, and 10% censoring, respectively. This is a reasonable result, because the random covariate approach involves fitting a quadratic Cox regression model, $\text{exp}\left(\gamma_{0}\mu_{j}+\sigma_{j}^{2}\gamma_{0}^{2}/2\right)$, instead of exp(*γ*_0_, *μ_j_*). The quadratic form takes into account the variance of each score, use of the compound score without acknowledgement of covariate error yields lower power.

**Figure 2:**
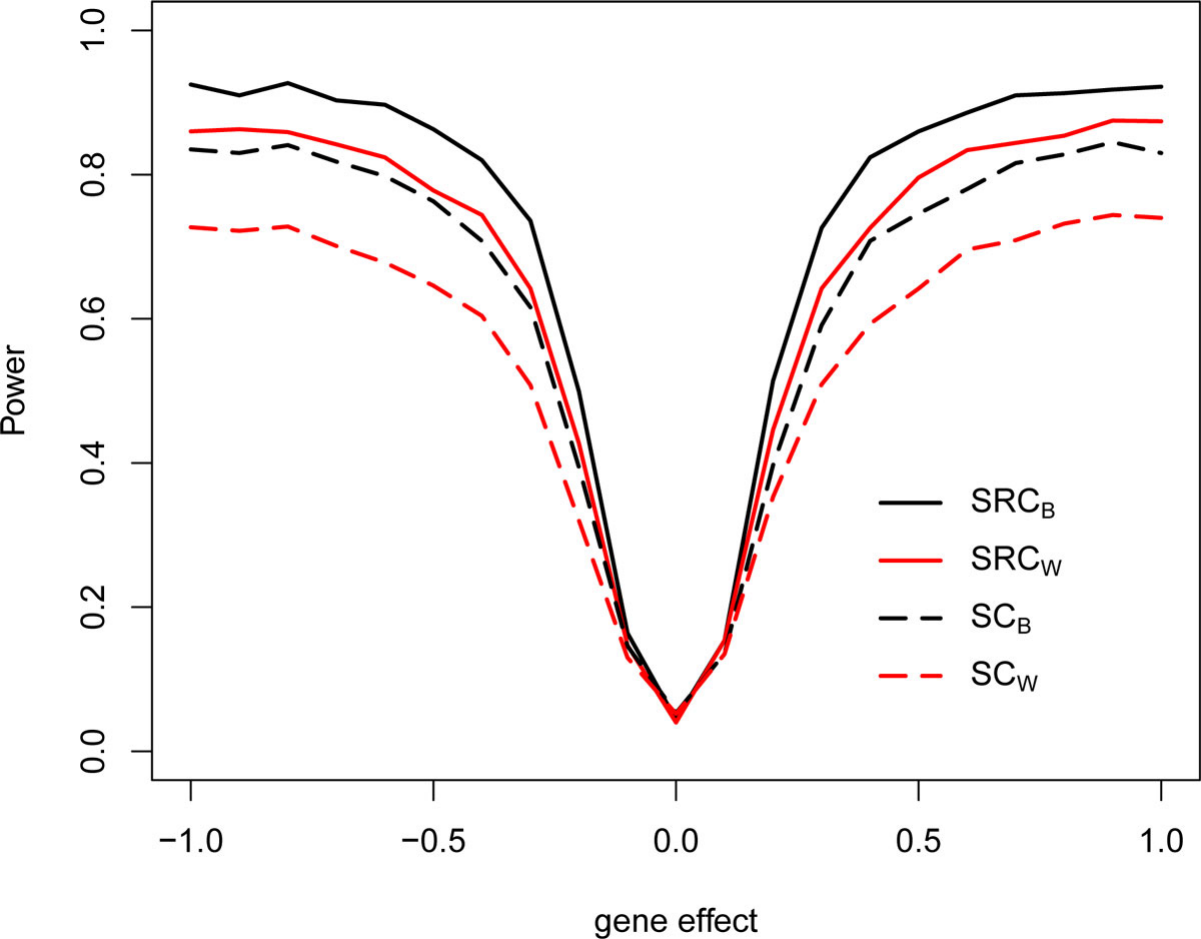
**Power curves with varying gene effect**.

To further illustrate the effect of treating the compound score as a random covariate, in Figure 2, we show the power curves of the *SRC_B_, SC_B_, SRC_W_*, and *SC_W _*methods for gene effect varying from -1 to 1. The total sample size was set to 100, and the censoring fraction was fixed at 50%. As shown in Figure 2, difference in power between *SRC_B _*and *SC_B _*and between *SRC_W _*and *SC_W _*increases as gene effect size increases, when gene effect size increases, the quadratic term can more accurately account for variance in the effect. Thus, treating the compound score as a random covariate in the Cox regression model provides greater power.

**Figure 3:**
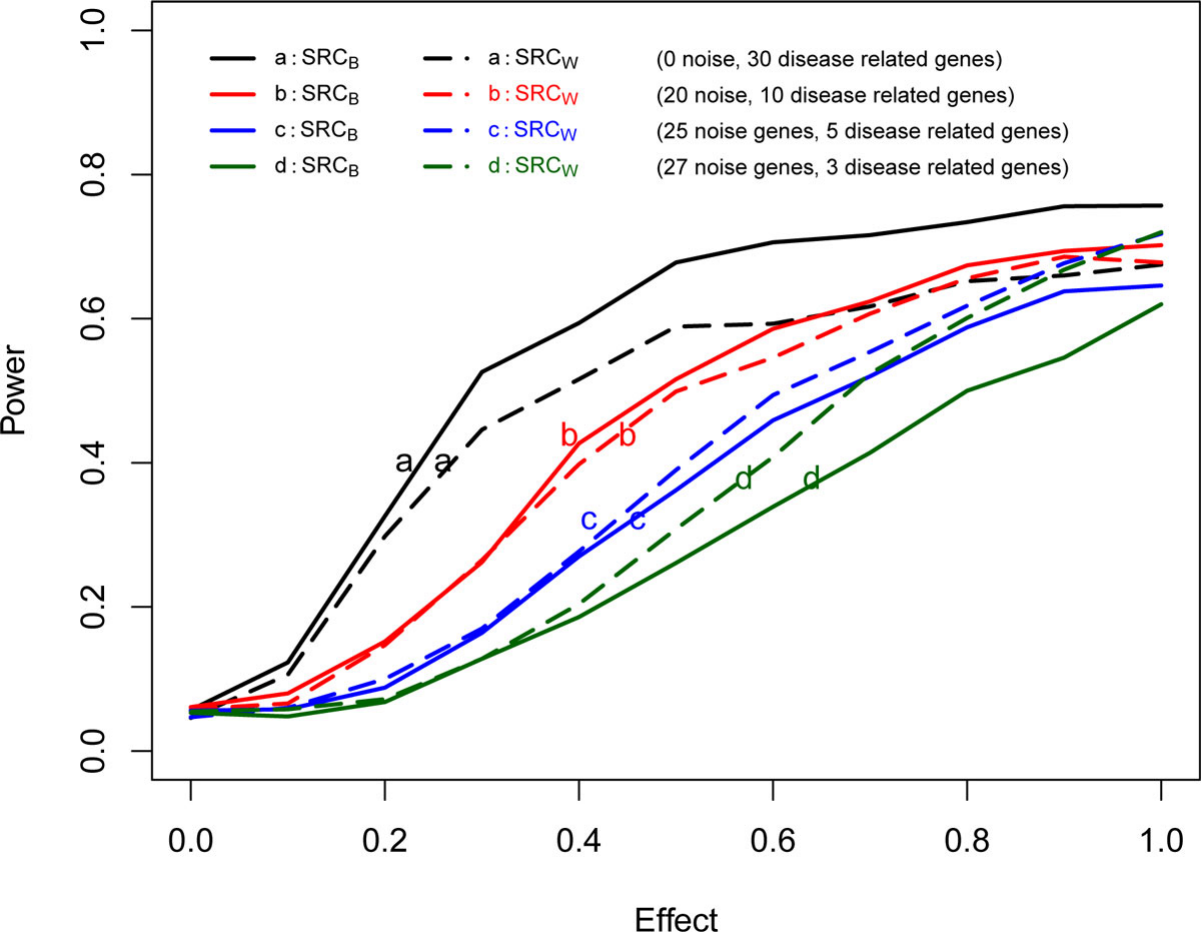
**Power curves with varying gene effect and number of noise genes (sample size, 50, censoring fraction, 10%)**.

For the first scenario, tests based on *SRC_B _*have higher power than those based on *SRC_W_*. In the second scenario, however, *SRC_W _*yields higher power than *SRC_B_*. This pattern change seems to relate to the increase in noise in the gene set. For Figure 3, we fixed the total number of genes at 30, sample size, 50; censoring fraction, 10% in one gene set, and show the power curves as gene effect and number of noise genes increase. There are eight lines in Figure 3. All solid lines indicate power curves for *SRC_B _*while all long-dash lines indicate power curves for *SRC_W_*. Situation *a *has 30 disease related genes, *b *has 10 disease related genes and 20 noise genes; *c *has 5 disease related genes and 25 noise genes, and *d *has 3 disease related genes and 27 noise genes. As gene effect increases, all powers increase. As the number of noise genes increases (from *a *to *d*), however, the powers of *SRC_B _*and *SRC_W _*decrease. With no noise genes (case *a*), the power of *SRC_B _*is always greater than that of *SRC_W_*. As the number of noise genes increases, however, the power of *SRC_W _*gradually improves over that of *SRC_B_*. This results from the fact that the compound covariate *SRC_W _*takes into account the variance of $\hat{\beta }$. Similar results were obtained with larger sample size (75, 100) and censoring fraction (30%, 50%). Consequently, if prior biological knowledge indicates many noise genes in a given gene set/pathway, we recommend use of the compound covariate *SRC_W _*over *SRC_B_*.

**Figure 4:**
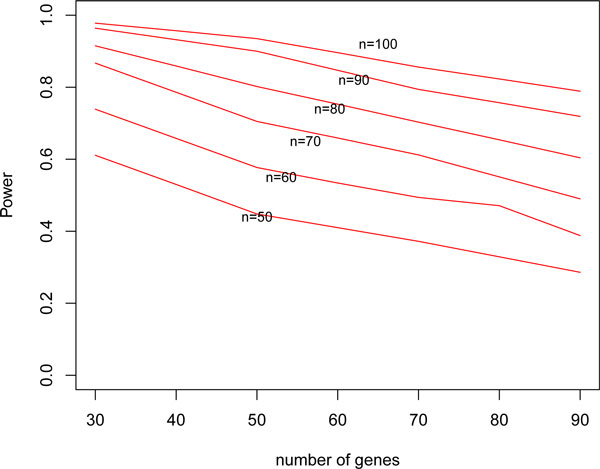
**Power curves with different numbers of genes and sample sizes**.

Figure 4 shows the power curve for *SRC_B _*with different sample sizes and different numbers of disease related genes. In this simulation design, the censoring fraction was fixed at 30%. The effect of all disease related genes was set to 0.5. As expected, the power increases as sample size grows. Power decreases, however, as the number of disease genes increase. Under this specified setting, if we need 80% power and have sample size 100, we can include about 90 disease related genes in this analysis. If we have sample size 60, however, we can only include fewer than 30 disease related genes. This result indicates the need for greater sample size to preserve power when a a gene set includes a large number of disease genes.

### Examples

In this section, we demonstrate our methodology using two examples, an Amsterdam 70-gene breast cancer gene signature [1] and a data set involving two pathways for non-small-cell lung cancer. All tests with nominal level 0.05 were applied to the training cohort. The R code for obtaining p-values for the proposed testing procedure is available from the authors upon request.

### Breast cancer data set

**Figure 5:**
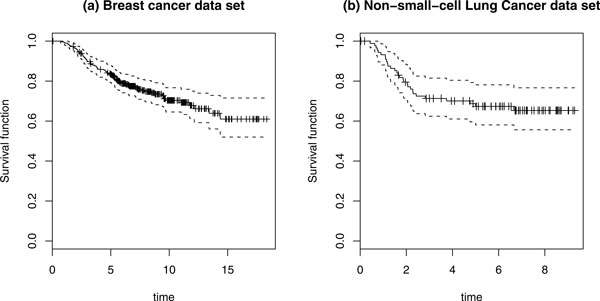
**Kaplan-Meier curves for two data sets**.

The well-known Amsterdam 70-gene breast cancer gene signature was published by Van't Veer [1]. To evaluate the previously established 70-gene prognosis file, Van de Vijver [16] further classified an additional 295 consecutive patients with stage I or II breast cancer to validate the breast cancer gene signature. Because the 295 patients are independent of the original data, we re-analyzed this data set using our methodology. In this data set, patients were followed for a median of 7.2 years, with 79 observed deaths. The survival curve is shown in Figure 5 (a) and the testing results, including estimated coefficients (Coef.), relative risk (RR), and p-values are given in Table 3.

**Table 3:** Breast cancer data set analysis

Method	Coef	RR	p-value
*SRC_B_*	0.052	1.12	1.9 × 10^-8^
*SRC_W_*	0.022	1.12	1.8 × 10^-8^
*SC_B_*	0.093	1.10	1.1 × 10^-7^
*SC_W_*	0.040	1.04	1.3 × 10^-7^
*DC_B_*	0.078	1.08	8.6 × 10^-13^
*DC_W_*	0.015	1.02	1.1 × 10^-13^

To evaluate the established 70 breast cancer gene signature published by Van't Veer with ther proposed method.

Although all coefficients and relative risks are very close, the p-values are very different. When using *DC_B _*and *DC_W_*, the p-values are 8.6 × 10^-13 ^and 1.1 × 10^-13^, respectively. When treating the compound covariate as fixed, the p-values of *SC_B _*and *SC_W _*are 1.1 × 10^-7 ^and 1.3 × 10^-7^. When using our procedure, the p-values of *SRC_B _*and *SRC_W _*are 1.9 × 10^-8 ^and 1.8 × 10^-8^. Although the results remain significant regardless of method, we achieve appropriate p-values for the training cohort, showing that the 70-gene prognosis signature can be used to evaluate early events in breast cancer patients. We get consistent conclusion with the other researches [17,16].

### Non-small cell lung cancer data set

We also tested our method by applying it to a publicly available non-small-cell microarray data set downloaded from National Center for Biotechnology Information Gene Expression Omnibus (GSE14814). There are 90 gene expression profiling conducted on mRNA isolated from frozen tumor samples. In this example, two well-known cancer-related pathways were used to test association with survival outcomes for demonstration purposes. The first signaling pathway is the p53 pathway, which is induced by a number of stress signals, including DNA damage, oxidative stress, and activated ontogenesis. The other pathway is the NOD-like receptor signaling pathway, which has been associated with an increased risk for the development of different types of cancer [18]. There are 61 genes in p53 pathway and 54 genes in NOD-like receptor signaling pathway, respectively. The median follow-up time of these patients was 5.4 years, and the number of observed deaths was 29. Figure 5 (b) shows the survival curve, and the test results are given in Table 4.

**Table 4:** Non-small-cell lung cancer data set analysis

Method	Pathway	Coef	RR	p-value	Overall p-value
*SRC_B_*	NOD	0.033	1.0013	0.59	0.236
	P53	0.037	1.0044	0.67	
*SRC_W_*	NOD	0.016	1.0063	0.37	0.358
	P53	0.001	1.0002	0.99	

*SC_B_*	NOD	0.077	1.08	0.36	0.432
	P53	0.015	1.01	0.90	
*SC_W_*	NOD	0.034	1.03	0.24	0.432
	P53	-0.01	0.99	0.74	

*DC_B_*	NOD	0.072	1.07	0.37	2.29 × 10^-6^
	P53	0.314	1.37	0.003	
*DC_W_*	NOD	0.019	1.02	0.21	1.85 × 10^-5^
	P53	0.055	1.06	0.006	

To evaluate the 90 gene expression profiling from National Center for Biotechnology Information Gene Expression Omnibus (GSE14814). The first signaling pathway is the p53 pathway. The other pathway is the NOD-like receptor signaling pathway.

To summarize all the information, two compound covariates were used. As shown, conventional Cox regression yields overall p-values that are strongly statistically significant (2.29 × 10^-6 ^for *DC_B _*and 1.85 × 10^-5 ^for *DC_W_*). When treating the compound score as a fixed covariate and using a split data set, however, the p-values of *SC_B _*and *SC_W _*become 0.432 for both. When treating the compound score as a random covariate, the p-values of *SRC_B _*and *SRC_W _*become 0.236 and 0.358, respectively. Such divergent p-values suggest that an inappropriate method may well lead to misleading results.

### Concluding remarks

In this paper, we focused on survival outcomes and proposed a feasible and correct method for testing the compound covariate to evaluate its association with survival outcomes for training cohort data. We have described the use of a random covariate, *SRC_B_*/*SRC_W_*, to achieve correct testing results for training cohort data and moderately improve power as compared to the use of *SC_B_*/*SC_W_*. Simulation study shows that our proposed method performs consistently better than *SC_B_*/*SC_W_*, because the quadratic term utilized in the *SRC_B_*/*SRC_W _*method takes into account error in the compound covariate. We further found that an increase in sample size improves power when there is a high proportion of censored data. If the gene set of interest includes noise genes, we suggest that the compound covariate *SRC_W _*is a better choice than *SRC_B_*; whether noise genes or non-functionally related genes are hidden in gene set is a judgment call for a geneticist. In addition, we contend that a flaw of biomedical papers concerned with such topics report an bias p-value based on flawed compound covariate analysis for the same training data set. In this paper, we use a well-known 2-fold concept, with one part of the data to built compound covariate and the remainder part for testing if there is association between survival outcomes and the score to ensure correct p-values in the training data set. Note that we need to check the proportional hazards assumption.

Our method can simultaneously test for more than one gene set in a training cohort data. More generally, this procedure can be applied not only for survival outcomes, but also for binary or continuous outcomes. The weighted flexible compound covariate method WFCCM [19], an extension of the compound covariate, also allows for use of the method of statistical analysis presented here. In addition, this method can easily be extended to consider the interaction between random covariates and clinical observed covariates, as

$$h\left(t|z,w\right)=h_{0}\left(t\right)E\left[\text{exp}\left(\gamma_{0}z+\gamma_{1}^{\text{T}}w+\gamma_{2}^{\text{T}}\left(zw\right)\right)\right]$$

using the same analysis procedure. The chosen weight $\hat{\beta }$ or ŵ  can be adjusted by the other clinical observed covariates in the proposed framework. Our method, however, cannot be used to test the interaction between two random covariates, because of the complexity of specifying the distribution for the interaction between two random covariates; this is an area worthy of further investigation. Another one potential practical concern of the proposed method is that sample size must not be too small, higher fractions of censored data create the need for further increased sample size. Similarly, to achieve high power when studying a large number of genes, greater sample size is needed. When studying a large number of genes, ignoring the covariance that exists between genes does not influence the type I error rate, however, taking the covariance into account may increase power. Further research is required to address these limitations. Note that the permutation test (e.g., [20]) might be another method to calculate an appropriate p-value for the training dataset, however, with the permutation test, weights are not easily adjusted by the other covariates. Even for a small gene set, this approach may appear too expensive in computer time.

## Competing interests

The authors declare that they have no competing interests.

## Authors' contributions

PFS developed the mathematical derivations, designed and performed the simulation, and drafted the manuscript. Xi and HC performed the experimence about microarray analysis and gave valuable advice related to the compound score issues. YS supervised the research and managed this project. All authors read and approved the final manuscript.

## Supplementary Material

Additional file 1 **The explicit forms of *a, b *and *c***. Additional file 1 is a PDF file which shows the explicit forms of *a, b *and *c*. Then, the score statistic can be derived. Click here for file

Additional file 2 **The derivation of variance for compound covariates**. Additional file 2 is a PDF file which shows the derivation of variance for compound covariates. Click here for file
