# Supplementary material for: Statistical aspects of omics data analysis using the random compound covariate
Source: BMC Syst Biol. 2012 Dec 17;6(Suppl 3):S11. doi: 10.1186/1752-0509-6-S3-S11 (PMC3524312; doi:10.1186/1752-0509-6-S3-S11)
Supplement: Additional file 1 — The explicit forms of a, b and c. Additional file 1 is a PDF file which shows the explicit forms of a, b and c. Then, the score statistic can be derived. [file 1752-0509-6-S3-S11-S1.pdf]

Additional file 1: The explicit forms of a, b and c

For simplicity, we ignore the subscript in the following derivation. Let $f(z)$ be the normal probability density function with mean μ and variance σ^2.

$$\begin{aligned} a =& E[\exp(\gamma_0 z + \boldsymbol{\gamma}_1^{\mathrm{T}}\mathbf{w})] = \exp(\boldsymbol{\gamma}_1^{\mathrm{T}}\mathbf{w}) \int \exp(\gamma_0 z) f(z) dz \\ =& \exp(\boldsymbol{\gamma}_1^{\mathrm{T}}\mathbf{w}) \int \exp(\gamma_0 z) \frac{1}{\sigma\sqrt{(2\pi)}} \exp\Big(\frac{-(z-\mu)^2}{2\sigma^2}\Big) dz \\ =& \exp(\boldsymbol{\gamma}_1^{\mathrm{T}}\mathbf{w} + \gamma_0\mu + \gamma_0^2\sigma^2/2) \end{aligned}$$

$$\begin{aligned} b =& \partial a/\partial \boldsymbol{\gamma} = \begin{bmatrix} \partial a/\partial \gamma_0 \\ \partial a/\partial \boldsymbol{\gamma}_1 \end{bmatrix} \\ =& \begin{bmatrix} (\mu + \sigma^2\gamma_0)\exp(\boldsymbol{\gamma}_1^{\mathrm{T}}\mathbf{w} + \gamma_0\mu + \gamma_0^2\sigma^2/2) \\ \mathbf{w}\exp(\boldsymbol{\gamma}_1^{\mathrm{T}}\mathbf{w} + \gamma_0\mu + \gamma_0^2\sigma^2/2) \end{bmatrix} \end{aligned}$$

and

$$c = \begin{bmatrix} \frac{\partial a}{\partial \gamma_0 \partial \gamma_0} & \frac{\partial a}{\partial \gamma_0 \partial \boldsymbol{\gamma_1}} \\ \frac{\partial a}{\partial \boldsymbol{\gamma_1} \partial \gamma_0} & \frac{\partial a}{\partial \boldsymbol{\gamma_1} \partial \boldsymbol{\gamma_1}} \end{bmatrix}$$

where

$$\begin{aligned} \frac{\partial a}{\partial \gamma_0 \partial \gamma_0} =& (\mu + \sigma^2 + \sigma^2\gamma_0)\exp(\boldsymbol{\gamma}_1^{\mathrm{T}}\mathbf{w} + \gamma_0\mu + \gamma_0^2\sigma^2/2), \\ \frac{\partial a}{\partial \gamma_0 \partial \boldsymbol{\gamma_1}} =& \mathbf{w}^{\mathrm{T}}(\mu + \sigma^2\gamma_0)\exp(\boldsymbol{\gamma}_1^{\mathrm{T}}\mathbf{w} + \gamma_0\mu + \gamma_0^2\sigma^2/2), \\ \frac{\partial a}{\partial \boldsymbol{\gamma_1} \partial \gamma_0} =& \mathbf{w}(\mu + \sigma^2\gamma_0)\exp(\boldsymbol{\gamma}_1^{\mathrm{T}}\mathbf{w} + \gamma_0\mu + \gamma_0^2\sigma^2/2), \\ \frac{\partial a}{\partial \boldsymbol{\gamma_1} \partial \boldsymbol{\gamma_1}} =& \mathbf{w}\mathbf{w}^{\mathrm{T}}\exp(\boldsymbol{\gamma}_1^{\mathrm{T}}\mathbf{w} + \gamma_0\mu + \gamma_0^2\sigma^2/2). \end{aligned}$$

Then, just plug in a, b and c into equation (3) and (4) provided in the manuscript. We can get the score test statistic.
