# Supplementary material for: Statistical aspects of omics data analysis using the random compound covariate
Source: BMC Syst Biol. 2012 Dec 17;6(Suppl 3):S11. doi: 10.1186/1752-0509-6-S3-S11 (PMC3524312; doi:10.1186/1752-0509-6-S3-S11)
Supplement: Additional file 2 — The derivation of variance for compound covariates. Additional file 2 is a PDF file which shows the derivation of variance for compound covariates. [file 1752-0509-6-S3-S11-S2.pdf]

Additional file 2: The derivation of variance for compound covariates

Because z_j follows a normal distribution with mean μ_j and variance σ_j^2, it is straightforward to derive $\text{Var}(z_j) = \sum_{k=1}^{p} x_{jk}^2 s_{\beta_k}^2$. In the following, we show that the compound score using Wald statistics as weight has the variance

$$\text{Var}(z_j) = \sum_{k=1}^{p} \text{Var}(x_{jk}\hat{w}_k \text{sign}(\hat{\beta}_k)) = 3\sum_{k=1}^{p} x_{jk}^2.$$

Because

$$\begin{aligned}\text{Var}(z_j) &= \sum_{k=1}^{p} x_{jk}^2 \{E[(\hat{w}_k \text{sign}(\hat{\beta}_k))^2] - E[(\hat{w}_k \text{sign}(\hat{\beta}_k))]^2\} \\ &= \sum_{k=1}^{p} x_{jk}^2 \{E(\hat{w}_k^2) - E[(\hat{w}_k \text{sign}(\hat{\beta}_k))]^2\},\end{aligned}$$

and Wald statistics, $\hat{w}_k$, follow a chi-square distribution with degree of freedom 1, $E(\hat{w}_k^2)$ of the first term equals 3. Also, the second term, $E[(\hat{w}_k \text{sign}(\hat{\beta}_k))]$, can be decomposed as

$$\begin{aligned}E(\hat{w}_k|\text{sign}(\hat{\beta}_k) = 1)Pr(\text{sign}(\hat{\beta}_k) = 1) &+ E(-\hat{w}_k|\text{sign}(\hat{\beta}_k) = -1)Pr(\text{sign}(\hat{\beta}_k) = -1) \\ &= \frac{E(\hat{w}_k|\hat{\beta}_k > 0)}{2} - \frac{E(\hat{w}_k|\hat{\beta}_k < 0)}{2} = 0.\end{aligned}$$

Therefore, $\text{Var}(z_j)$ equals $3\sum_{k=1}^{p} x_{jk}^2$. It should be noted that, for simplicity, we treat $\hat{\beta}_i$ and $\hat{\beta}_j$ are independent for $i \neq j$. This can be extended in the future.
